# Introducing the forearm fracture index to define the diametaphyseal junction zone through clinical evaluation in a cohort of 366 diametaphyseal radius fractures

**DOI:** 10.1007/s00402-024-05664-0

**Published:** 2025-01-07

**Authors:** Christoph von Schrottenberg, Ricardo Beck, Susann Marie Beck, Christian Kruppa, Matthias Kuhn, Philipp Schwerk, Guido Fitze, Jurek Schultz

**Affiliations:** 1 Department of Pediatric Surgery, Faculty of Medicine and University Hospital Carl Gustav Carus, TUD Dresden University of Technology, Fetscherstraße, 74, 01307 Dresden, Germany; 2 Institute for Medical Informatics and Biometry, Faculty of Medicine, Technical University Carl Gustav Carus, Dresden, Germany

**Keywords:** Diametaphyseal junction zone, Distal radius fracture, Diametaphyseal radius fracture, Metadiaphyseal, TEPIK

## Abstract

**Background:**

Unstable diametaphyseal radius fractures (DMRFs) can be prone to complications, and treatment strategies are heterogeneous. Studies are difficult to interpret as definitions of the diametaphyseal junction zone (DMJZ) are impractical for clinical use, imprecise, or prone to error.

**Methods:**

We introduce the forearm fracture index (FFI) to define DMRFs in radiographs and ultrasound. The FFI is calculated by the ratio of the fracture’s distance to the distal radius growth plate over the width of the radius growth plate. The higher the FFI, the more proximal the fracture is. We define DMRFs to have an FFI between 1 and 2. All DMRFs treated at our institution between 2010 and 2020 were identified, and demographic data, fracture characteristics, and therapeutic strategies were assessed retrospectively. Comparative sub-analysis was performed between DMRFs(−) as defined in previous publications (Lieber in Unfallchirurg 114:292–299, 2011) and DMRFs( +) that were more proximal but still met our criteria.

**Results:**

516 DMRFs were identified, representing 13.0% of all screened radius fractures. Excluding buckle fractures and patients lost to follow-up, 366 DMRFs were eligible for further analysis. Conservatively managed DMRFs were more distal than those managed operatively, represented by a lower FFI (1.28 vs. 1.34, p = 0.0051). 21 (5.7%) of all DMRFs were identified as DMRFs( +). These were significantly more dislocated and necessitated surgery more often than DMRFs(−) (52.4 vs. 24.6%, p = 0.009).

**Conclusions:**

The FFI may be a good tool to identify and describe DMRFs. It can help guiding treatment decisions and make future studies on this entity more comparable.

**Level of evidence:**

Study of Diagnostic Test, Level II.

**Supplementary Information:**

The online version contains supplementary material available at 10.1007/s00402-024-05664-0.

## Introduction

**Fig. 1:**
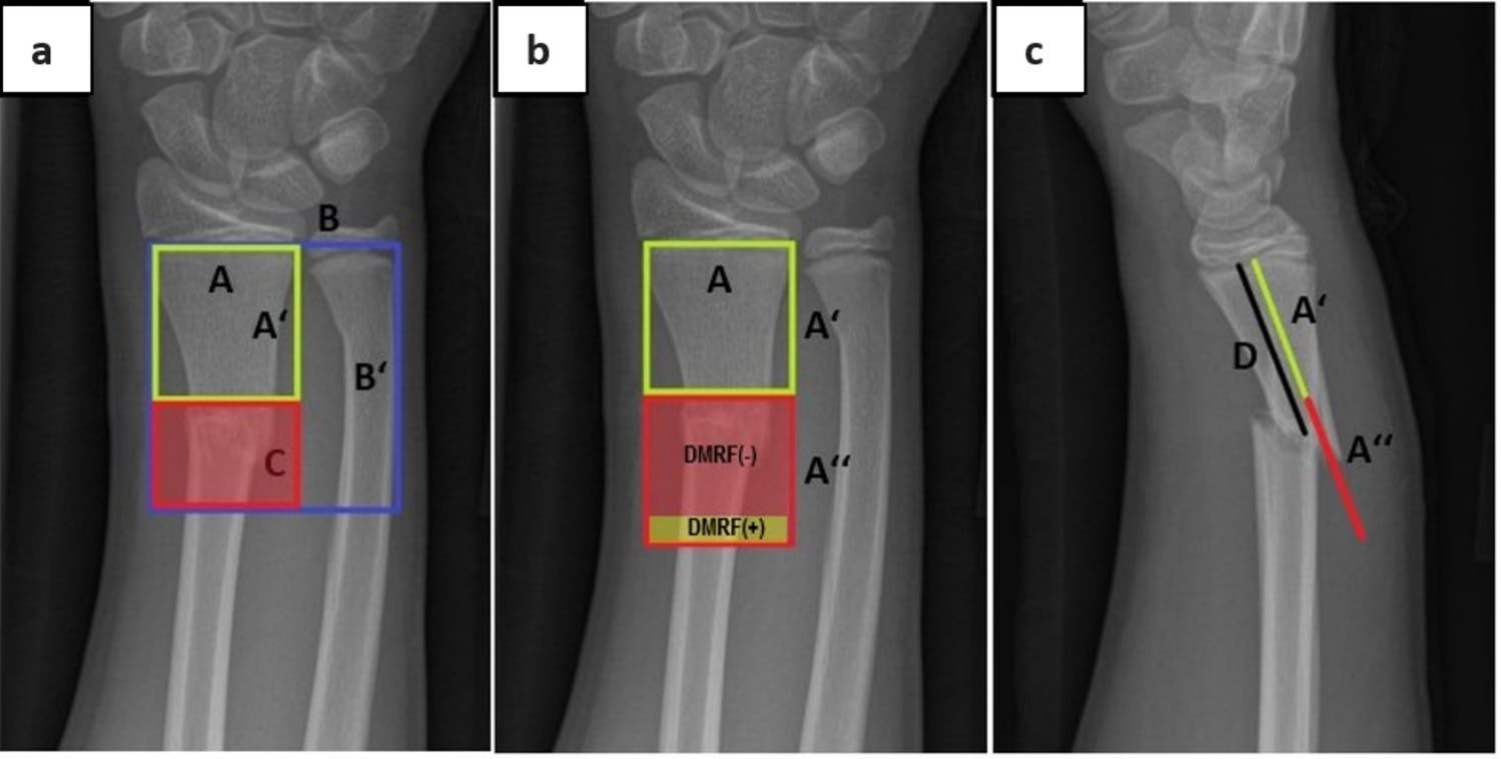
**a** Anterior–posterior radiograph (a.p.) of the right distal forearm of a 12-year-old patient with a complete diametaphyseal radius fracture (DMRF); A marks the width of the growth plate of the radius; B marks the combined width of the growth plate of radius and ulna; C marks the diametaphyseal junction zone (DMJZ) as defined by Lieber et al.; **b** a.p. radiograph of the right distal forearm of a 12-year-old patient with a complete DMRF; A’’ marks the DMJZ as defined by our group; DMRFs as defined by Lieber et al. are located within the red rectangle and marked DMRF(-); DMRFs as defined by our group but proximal to DMRF(-) are located within the orange rectangle and are marked DMRF( +); **c** Lateral radiograph of the right distal forearm of a 12-year-old patient with a complete DMRF; D marks the distance from the fracture line to the growth plate; the ratio of D over A defines the forearm fracture index (FFI). DMRFs have an FFI > 1 and ≤ 2

Most pediatric fractures affect the upper limb, with 36–40% involving the radius or the ulna [2–5]. The distal forearm is the most frequent location of injury, with 19–33% of all fractures occurring there [2, 5]. However, literature on the incidence of fractures in the diametaphyseal junction zone (DMJZ) is scarce [6]. Unstable diametaphyseal radius fractures (DMRFs) are a matter of great interest to pediatric surgeons. Loss of reduction, refractures, and limited remodeling can make the treatment challenging [7–10]. The AO Pediatric Comprehensive Classification of Long Bone Fractures (PCCF) disregards fractures within the DMJZ as an own entity. It defines the metaphysis as a square over the growth plates of the radius and ulna (Fig. 1a). The area proximal to this square is the diaphysis. The PCCF follows anatomical structures and has little predictive value concerning treatment strategies or prognosis [11–14]. Existing definitions of the DMJZ are inconsistent, making it difficult to compare studies and surgical techniques.

Lieber et al. defined the DMJZ as the part of the metaphysis proximal to the square over the radius growth plate alone (Fig. 1, a) [1]. Another definition by Kim et al. requires multiple variables as DMRFs are defined as “fracture[s] with (1) the distance between the fracture line and the distal articular surface between 35 and 60 mm; (2) the ratio of the length of distal fragment to the total length of radius within 25%; and (3) the ratio of the maximal diameter at 2 cm proximal to the fracture line to that at 2 cm distal to the fracture line within 70%” [15]. A third definition characterizes the DMJZ as the distal third of the radius minus the square of the width of the radius growth plate [16]. It is difficult to use the latter two definitions in clinical practice as most radiographs do not show the entire forearm to reduce radiation exposure; hence, the distal third of the radius cannot be defined in many cases. Moreover, these classifications provide little prognostic information. To provide help in selecting a therapeutic strategy for DMRFs, a recent study advocates dividing the DMJZ as defined by Lieber et al. into a proximal, an intermediate, and a distal third [1, 17].

To take this idea further and to overcome the aforementioned difficulties, we introduce the forearm fracture index (FFI), a measure to define and further locate DMRFs on radiographs and ultrasound. Advantages and disadvantages of this classification will be outlined, including its applicability to ultrasound which has become increasingly important in diagnosing distal radius fractures [18–21]. Finally, fracture characteristics and therapy strategies in a cohort of 366 patients with DMRFs will be assessed, and subgroup analysis will be performed between DMRFs(-) defined according to Lieber et al. and fractures that were located more proximally but still considered DMRFs( +) as defined by our group.

## Materials and methods

### Introducing the forearm fracture index and defining the diametaphyseal junction zone

The FFI was calculated as follows: the ratio of the fracture’s distance to the radius growth plate (D) in the lateral radiograph over the radius growth plate’s width (A) in the anterior–posterior (a.p.) radiograph (Fig. 1c).


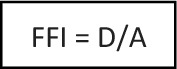


If this ratio is between 1 (distal limit) and 2 (proximal limit), the fracture is termed a DMRF. Fractures of the radius could hence be categorized as:

- Epiphyseal—for these fractures, the FFI does not apply;
- Metaphyseal—these fractures have an FFI of < 1;
- Diametaphyseal—these fractures have an FFI of > 1 and ≤ 2;
- Diaphyseal—these fractures have an FFI of > 2;
- Proximal—for these fractures, the FFI does not apply;

### Clinical evaluation of the new definition of the DMJZ

We retrospectively analyzed all forearm fractures in patients aged 16 years or younger treated at our institution from 2010 to 2020. This study was approved by our local ethics committee (EK 433102016). Data was retrieved using ICD-Codes S52.0–S52.9. Duplicates were eliminated. Further exclusion criteria were falsely coded fractures, solitary fractures of the ulna, pathological fractures, and closed growth plates in the initial radiograph. Buckle fractures and fractures lost to follow-up were included in the categorization of radius fractures but were excluded from further analysis. A patient flow diagram is provided in the Supplementary Material (Figure S1) [22].

The FFI was calculated as described above and all DMRFs (FFI > 1 and ≤ 2) were further analyzed. Demographic and clinical data were collected and, using IMPAX EE Version R20 XIX (AGFA HealthCare, Mortsel, Belgium), the following geometric parameters and variables were assessed in initial radiographs and during follow-up: the angulation in degree, the translation in percentage of the shaft’s width at the level of the fracture, the angle of the fracture line in degree and whether a shortened fracture was present. A comparative sub-analysis of fracture characteristics and therapy strategies was performed between fractures that met Lieber et al.’s criteria, termed DMRFs(−), and those that were defined as diaphyseal fractures by Lieber et al. but still considered a DMRF by our definition, termed DMRFs( +) [1].

Comparison of metric data was performed using the t-test with Welch’s correction, and results were displayed as mean values with standard deviation when data was normally distributed. The Mann–Whitney-U-test was used when data was not normally distributed, and results were displayed as median values with interquartile ranges. Categoric data was compared using the Fisher exact test or the Chi-Square test when applicable. Data curation and statistical analysis were performed using GraphPad Prism version 8.4.3 for Windows, GraphPad Software, San Diego, California, USA (www.graphpad.com).

## Results

### Categorization of all radius fractures using the forearm fracture index

**Fig. 2:**
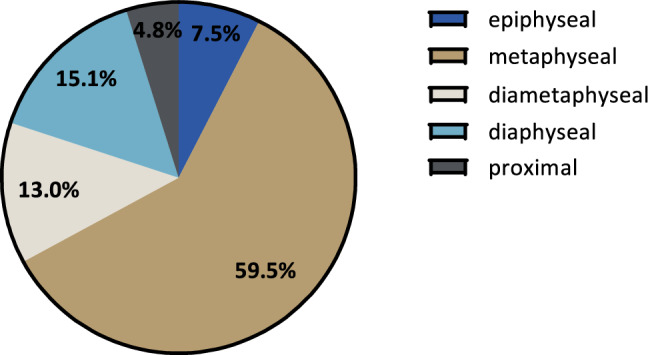
Distribution of localization of 3956 radius fractures in patients ≤ 16 years of age admitted to our tertiary pediatric trauma center from 2010 to 2020

3956 radius fractures were identified. Of these, 59.5% affected the distal metaphysis, 15.1% the diaphysis, 7.5% the distal epiphysis and 4.8% were proximal, the majority of which were fractures of the radial neck. The remaining 516 (13.0%) were fractures of the DMJZ (Fig. 2). Of these, 132 (25.6%) were buckle fractures and 18 (3.5%) patients were lost to follow-up. After exclusions, 366 DMRFs in 366 patients (262 male, 104 female; p < 0.0001) were available for further analysis. The median age at the time of the accident was 8 years (IQR, 6–11 years).

### Applicability of the forearm fracture index in ultrasound

**Fig. 3:**
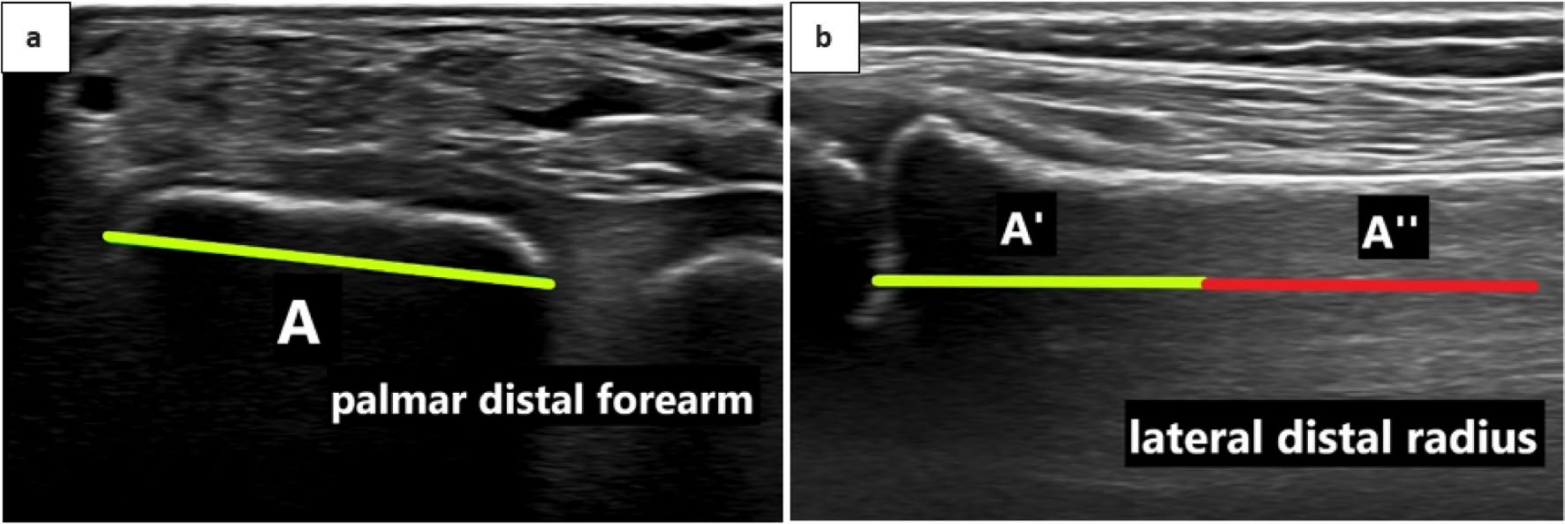
**a** Transverse ultrasound of the palmar distal forearm; A marks the width of the radius at the level immediately proximal to the growth plate which approximates the width of the radius growth plate; **b** Longitudinal ultrasound of the lateral distal radius; A’ marks the width of the radius growth plate as determined in the transverse ultrasound and is applied twice to determine the diametaphyseal junction zone A’’

To define the DMJZ using ultrasound, an assessment of the radius growth plate’s width is necessary. This can be done by holding a linear transducer orthogonally to the patient’s palmar forearm perpendicular to the radius axis. Sliding towards the wrist joint, the radius shaft must be in the center of the screen. Towards the growth plate, the diameter of the radius shaft increases continuously until the growth plate appears and the echogenic cortex disappears. The width of the radius metaphysis just proximal to where the cortex disappears approximates the width of the radius growth plate (Fig. 3). The growth plate itself is hypoechogenic, lacking the clear soft-tissue-to-bone interface that characterizes skeletal ultrasound.

### Fracture characteristics of 366 diametaphyseal radius fractures and comparison between DMRFs(-) and DMRFs( +)

Median radiologic follow-up lasted 38 days (IQR, 29.4–60 days). The mean FFI of the cohort was 1.29 ± 0.22 (range, 1.01–1.97). Of the 366 DMRFs included, 345 were identified as DMRFs(−) [23]. 21 fractures were identified as DMRFs( +), as their FFI was between 1 and 2 but proximal to the area defined by Lieber et al. The FFI differed significantly between DMRFs(−) and DMRFs( +) (1.26 ± 0.19, [range, 1.01–1.87] vs. 1.77 ± 0.15, [range, 1.42–1.97]; p < 0.0001). Dividing our cohort into distal, intermediate, and proximal DMRFs using the FFI resulted in 65.8% distal, 26.5% intermediate, and 7.7% proximal DMRFs [17]. 220 (60%) of all DMRFs were complete, while 40% were greenstick fractures. This rate did not differ between DMRFs(−) and DMRFs( +). Median angulation in the a.p. radiograph was 6° (IQR, 2–11°) and was significantly increased in DMRFs( +) compared to DMRFs(−) (12 vs. 6°, p = 0.0003). Median angulation in the lateral radiograph was 17° and did not differ between the two groups (17 vs. 20°, p = 0.309).

132 fractures (36.1%) presented with a translation in the a.p. radiograph with a median dislocation of 25% of the shaft’s width (IQR, 17–46%). There was no statistically significant difference in the incidence (36.2 vs. 33.3%, p > 0.9999) or the severity (25% [IQR, 17–44%] vs. 40% [IQR, 18–55%]; p = 0.389) of translation in the a.p. radiograph between DMRFs(−) and DMRFs( +). 107 fractures (29.2%) presented with a translation in the lateral radiograph with a median dislocation of 100% (IQR, 40–100%) of the shaft’s width. There was no significant difference in the incidence (30.1 vs 14.3%, p = 0.1434) or the severity (100% [IQR, 36–100%] vs. 100% [IQR, 100–100%]; p = 0.2055) of translation in the lateral radiograph between patients with DMRFs(−) and DMRFs( +). The overall median angles of the fracture line in the a.p. and lateral radiographs were 8° and 14°, respectively. These angles differed significantly between DMRFs(-) and DMRFs( +) (a.p.: 7 vs. 17°, p = 0.0112; lateral: 13 vs. 33°, p < 0.0001). 24 DMRFs (6.6%) were oblique fractures with a fracture line angle in the a.p. radiograph of > 30°. The rate of oblique fractures was significantly increased in the group of DMRFs( +) compared to DMRFs(-) (33.3 vs. 4.9%, p = 0.0001). The rate of shortened fractures was 20.2% and did not differ between the two groups. All fracture characteristics are displayed in Table S1 of the Supplementary Materials.

### Therapy strategies in DMRFs(−) and DMRFs( +) with regard to the FFI

In this study’s cohort of 366 DMRFs, 270 fractures (73.8%) were managed conservatively, 215 of which required reduction initially, which was performed under analgosedation in the outpatient clinic/emergency ward. 9 conservatively managed fractures (3.3%) were immobilized in a forearm cast; all others were immobilized in an upper arm cast. 96 patients (26.2%) underwent surgery under general anesthesia. The preferred osteosynthesis was transepiphyseal percutaneous intramedullary Kirschner-wire fixation (TEPIK) as described by our group previously and was performed in 56 patients (58.3%) [24]. Bicortical Kirschner-wire (K-wire) fixation via the radial styloid process or the radial metaphysis proximal to the zone of Ranvier was performed in 16 patients (16.7%) [25, 26]. Elastic stable intramedullary nailing (ESIN) was used in 19 patients (19.8%), and 5 patients (5.2%) received plate osteosynthesis [27–31].

Osteosynthesis was performed significantly more often in DMRFs( +) than in DMRFs(−) (52.4 vs. 24.6%, p = 0.009). The preferred osteosynthesis for DMRFs(−) was TEPIK (61.2%), while for DMRFs( +), ESIN osteosynthesis was performed more frequently (63.6%). Table 1 gives an overview of the different therapy strategies.

**Table 1:** Different therapeutic approaches for diametaphyseal radius fractures

	cohort (n = 366)	DMRF(-) (n = 345)	DMRF( +) (n = 21)	p
Conservative	270 (73.8%)	260 (75.4%)	10 (47.6%)	0.009
*Forearm cast	9 (3.3%)	9 (3.5%)	–	–
*Upper arm cast	261 (96.7%)	251 (96.5%)	10 (100%)	> 0.9999
Operative	96 (26.2%)	85 (24.6%)	11 (52.4%)	0.009
**TEPIK-osteosynthesis	56 (58.3%)	52 (61.2%)	4 (36.4%)	0.192
**Bicortical Kirschner-wire fixation	16 (16.7%)	16 (18.8%)	–	–
**ESIN-osteosynthesis	18 (18.8%)	11 (12.9%)	7 (63.6%)	0.0006
**Plate-osteosynthesis	5 (5.2%)	5 (5.9%)	–	–
Outpatient treatment	254 (69.4%)	244 (70.7%)	10 (47.6%)	0.0476

Frequencies of the different therapeutic approaches for diametaphyseal radius fractures (DMRFs) in our cohort of 366 patients and comparison between DMRFs(-) and DMRFs( +); *percentages refer to all patients treated conservatively in the respective column; **percentages refer to all patients treated operatively in the respective column; FFI, forearm fracture index; TEPIK, transepiphyseal percutaneous intramedullary Kirschner-wire osteosynthesis; ESIN, elastic stable intramedullary nailing

**Fig. 4:**
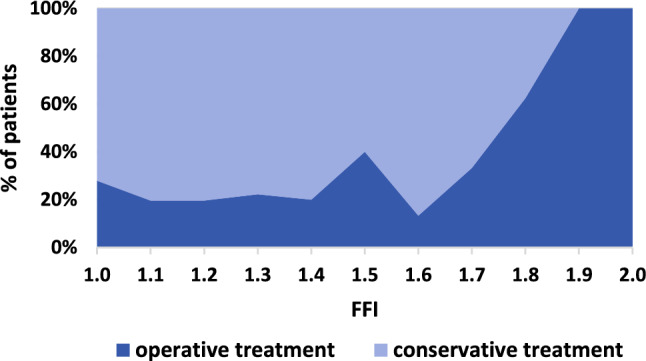
Conditional density plot shows percentages of operatively and conservatively treated diametaphyseal radius fractures (DMRFs) depending on the forearm fracture index (FFI) in 366 patients admitted to our tertiary pediatric trauma center between 2010 and 2020

Conservatively treated DMRFs had a significantly smaller FFI than operatively treated DMRFs (1.27 ± 0.22 [range, 1.01–1.89] vs. 1.34 ± 0.28 [range, 1.01–1.97]). Figure 4 visualizes treatment strategies (operative vs. conservative) in co-occurrence with the FFI.

**Fig. 5:**
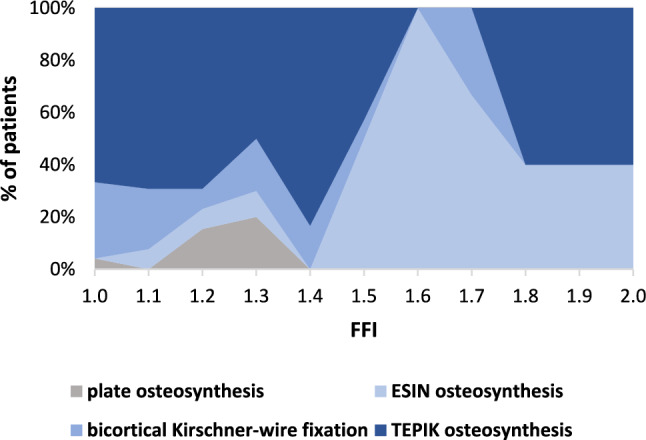
Conditional density plot shows percentages of various techniques of osteosynthesis depending on the forearm fracture index (FFI) in 96 surgically managed diametaphyseal radius fractures (DMRFs) admitted to our tertiary pediatric trauma center between 2010 and 2020; ESIN, elastic stable intramedullary nailing; TEPIK, transepiphyseal percutaneous intramedullary Kirschner-wire fixation

DMRFs managed with TEPIK had an FFI of 1.30 ± 0.27 (range, 1.01–1.97), bicortical K-wire fixated DMRFs had an FFI of 1.22 ± 0.22 (range, 1.02–1.76), while those managed with ESIN had an FFI of 1.61 ± 0.22 (range, 1.18–1.92). Finally, DMRFs managed with plate osteosynthesis had an FFI of 1.24 ± 0.13 (range, 1.03–1.38). The co-occurrence between the FFI and the different surgical techniques is displayed in Fig. 5.

## Discussion

### Introducing the forearm fracture index and defining the diametaphyseal junction zone

Applying the FFI to identify DMRFs, we have found an incidence for DMRFs of 13.0% of all radius fractures. This is in line with the recent literature reporting 16.1% of all forearm fractures to be DMRFs [17]. These results indicate that DMRFs are not rare and might represent a relevant challenge for pediatric traumatologists. As mentioned above, existing definitions of DMRFs bear several weaknesses. For some, radiographs of the entire forearm are necessary, which conflicts with the principle of limited radiation exposure [15, 16]. Anatomic variants such as ulna minor variants or twisted radiographs may lead to imprecise values when the growth plate’s width of radius and ulna combined need to be measured [1]. Furthermore, applying this distance to the radius in the a.p. radiograph to define the DMJZ may lead to false results in the case of a shortened fracture or an angulation in the lateral radiograph, which represent the most common displacements. In these cases, the distance from the fracture line to the radius growth plate will appear shorter than it actually is. This may lead to fractures being misinterpreted as metaphyseal [1]. An example is visualized in Figure S2 of the Supplementary Material.

Another disadvantage of existing definitions is their limited value in describing the exact location of the fracture, which is essential for choosing the right therapy strategy. Stark et al. tried to overcome this by categorizing DMRFs as defined by Lieber et al. into proximal, intermediate, and distal ones [17, 23]. Instead, we present the FFI as a continuous, quantitative variable that clearly distinguishes between diaphyseal and diametaphyseal radius fractures while unambiguously describing the exact fracture location within the DMJZ. Using the FFI to define the DMJZ may eliminate some sources of error. Measuring only the width of the radius growth plate might be easier than measuring the radius and ulna growth plates together. As the fracture’s distance to the growth plate is assessed in the lateral radiograph or with sonography, it is independent of angulation in the lateral radiograph and any potential shortening of the fracture. In case of angulation in the a.p. radiograph, the distance can be assessed in the a.p. radiograph or with ultrasound.

Nevertheless, in the rare case of severe angulation both in the a.p. and the lateral radiograph, the FFI cannot be determined reliably. In cases of severe displacement, it may seem reasonable to take only one radiograph to indicate surgery. Consequently, the radiograph of the second plane can be spared in the emergency ward, which might hinder determining the FFI preoperatively as well.

Our definition of the DMJZ (FFI > 1 and ≤ 2) includes a slightly longer part of the radius than the definition offered by Lieber et al., thus including 21 of 366 (5.7%) fractures that would have been formerly classified as diaphyseal [1]. Even though this is a small percentage, this group of proximal DMRFs( +) is particularly important to be accounted for. DMRFs( +) present with an increased angulation in the a.p. radiograph (12 vs. 6°, p = 0.0003), which is the direction of dislocation less likely to correct spontaneously [7, 9, 10]. Interestingly, in our cohort, the overall incidence of oblique DMRFs with a fracture line angle of > 30° in the a.p. radiograph was 6.6%, notably more than in the cohort analyzed by Stark et al., who described this in only one patient (1.1%) [17]. Furthermore, the angles of the fracture line both in the a.p. and the lateral radiographs were significantly increased in DMRFs( +) (a.p.: 17 vs. 7°, p < 0.0112; lateral: 33 vs 13°, p < 0.0001), possibly making them more prone to secondary dislocation. Consequently, DMRFs( +) were treated operatively significantly more often (52.4 vs. 24.6%, p = 0.009).

### Correlating the choice of osteosynthesis with the exact localization of the DMRF

Among various techniques, the optimal therapeutic strategy for DMRFs might depend on their exact localization within the DMJZ [1, 17]. Accordingly, conservatively treated DMRFs in our cohort had a lower FFI than those treated operatively (1.27 vs. 1.34; p = 0.005). Stark et al. divided their retrospectively analyzed cohort of 88 DMRFs into distal (47.7%), intermediate (29.5%), and proximal (22.7%) DMRFs and found proximal DMRFs to be treated mainly with ESIN or intramedullary K-wire-osteosynthesis. This tendency is also found in our cohort since ESIN osteosynthesis was most frequent in proximal DMRFs with a mean FFI of 1.61 compared to the overall average of 1.27 (p < 0.0001). DMRFs managed with TEPIK (FFI = 1.30) or bicortical K-wire fixation (FFI = 1.22) were rather distal. In comparison, the preferred surgical technique for distal DMRFs in the cohort analyzed by Stark et al. was bicortical K-wire fixation. However, in our cohort the variability of surgical techniques decreased over the study period as TEPIK became the favored technique in all unstable DMRFs after its establishment in 2010. While Lieber et al. describe the styloid process as the preferred entry point for TEPIK osteosynthesis, our entry point for the K-wire lies distally to Lister’s tubercle, which offers excellent results with no secondary dislocation. Metal removal was performed after 4 weeks without any anesthesia [23, 24].

Multiple surgical approaches for DMRFs, including various techniques for intramedullary K-wire or ESIN osteosynthesis such as the antegrade intramedullary nailing of the radius, have been published as of today [8, 23, 24, 32, 33]. While most of these techniques have not been universally accepted, antegrade intramedullary nailing of the radius seems to become more and more popular due to its good results and low risk of complications [15, 34–36]. One disadvantage of ESIN osteosynthesis still remains as metal removal must be performed under a second general anesthesia. Still, immobilization in a plaster cast until metal removal poses a disadvantage of TEPIK against ESIN osteosynthesis.

The FFI could be used to compare different surgical techniques better, as it clearly categorizes and specifies DMRFs, thus helping future trials.

### Assessing the FFI with ultrasound

Ultrasound has become an invaluable tool in diagnosing distal radius fractures in children as it is free of radiation, can be learned quickly, has a high degree of specificity and sensitivity, and may increase the comfort for pediatric patients as the diagnostic setting can be framed child-friendly more easily [18–21]. Unfortunately, previously published definitions of the DMJZ cannot be used in ultrasound as it is not feasible to determine the length of the entire radius [15, 16]. Also, the definition provided by Lieber et al. can be challenging to apply to ultrasound images since the limited width of many linear transducers hinders the correct visualization of the ulna and radius growth plates simultaneously. To determine the FFI in ultrasound, one only needs to depict the radius growth plate, which can be assessed quickly.

In some cases, the applicability of ultrasound to diagnose distal radius fractures may not be feasible due to severe dislocation [37]. In these cases, the FFI cannot be determined via ultrasound.

### Limitations

This study's limitations include its retrospective, single-center design. With regard to the rate of surgically managed DMRFs, a certain bias cannot be excluded as our institution is a tertiary pediatric trauma center, hence complex fractures necessitating surgery are often referred to us from peripheral institutions. However, to our knowledge, this is the largest cohort of patients with DMRFs systematically analyzed. Furthermore, no standardized protocol for the choice of the osteosynthesis was followed. During the study period, the likelihood of choosing TEPIK osteosynthesis increased because of the good results that were observed by our group using this technique [24].

## Conclusion

With a cohort of 366 patients, this is an extensive retrospective analysis of DMRFs in children and adolescents. We have introduced the FFI, a simple tool to define DMRFs with an FFI between 1 and 2. It was shown that the choice of various surgical approaches for DMRFs depends on their localization, which can be expressed precisely using the FFI. We propose that the FFI can be assessed by ultrasound as measuring the width of the radius growth plate can be learned quickly. The FFI may help make future studies on DMRFs more comparable as it offers an objective, quantifiable tool to define DMRFs and to differentiate within the group of DMRFs. Prospective, randomized controlled multi-center trials are needed to determine a standardized surgical approach for various DMRFs stratified using the FFI.

## Supplementary Information

Below is the link to the electronic supplementary material.

Supplementary file1 (DOCX 366 KB).

## Data Availability

The data presented in this study are available on request from the corresponding author.

## Abbreviations

a.p.: Anterior–posterior
DMJZ: Diametaphyseal junction zone
DMRF: Diametaphyseal radius fractures
ESIN: Elastic stable intramedullary nailing
FFI: Forearm fracture index
IQR: Interquartile range
K-wire: Kirschner-wire
PCCF: AO pediatric comprehensive classification of long bone fractures
TEPIK: Transepiphyseal percutaneous intramedullary Kirschner-wire
