# Supplementary material for: Introducing the forearm fracture index to define the diametaphyseal junction zone through clinical evaluation in a cohort of 366 diametaphyseal radius fractures
Source: Arch Orthop Trauma Surg. 2025 Jan 7;145(1):115. doi: 10.1007/s00402-024-05664-0 (PMC11706922; doi:10.1007/s00402-024-05664-0)
Supplement: Supplementary file 1 — Supplementary file1 (DOCX 366 KB). [file 402_2024_5664_MOESM1_ESM.docx]

**Supplementary Materials**

(Lieber et al., 2010)


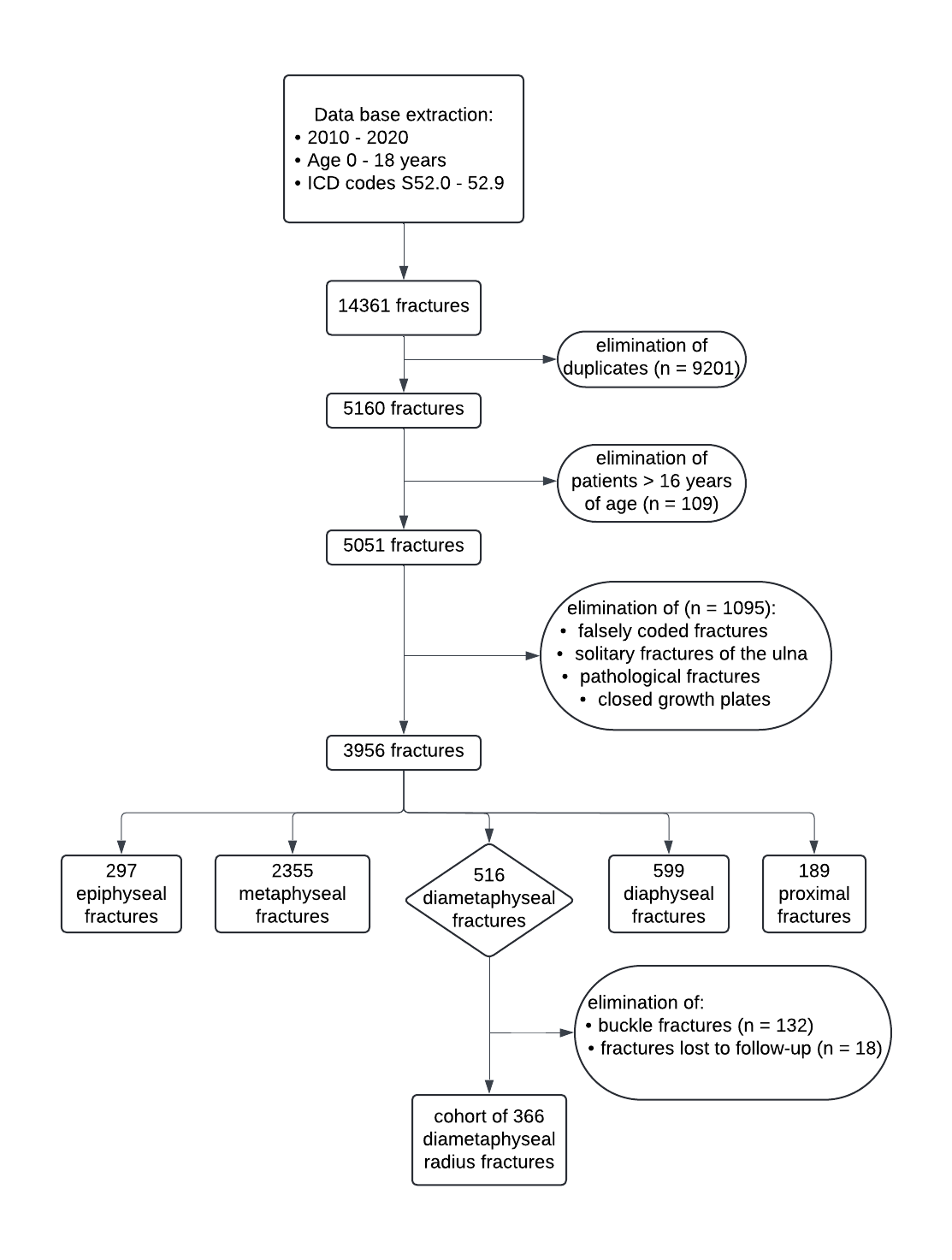


**Figure S1.** Patient Flow Diagram according to STARD 2015 guidelines (Cohen et al., 2016);

**Table S1.** Demographic data and fracture characteristics of our cohort of 366 patients with diametaphyseal radius fractures.

|  | cohort  (n = 366) | DMRFs(-)  (n = 345) | DMRFs(+)  (n = 21) | p** |
| --- | --- | --- | --- | --- |
| age (years) | 8 (6,11) | 8 (6,11) | 8 (5,12) | 0.7806 |
| male | 262 (71.6%) | 249 (72.2%) | 13 (61.9%) | 0.3240 |
| left-sided | 205 (56%) | 193 (56%) | 12 (57%) | > 0.9999 |
| greenstick fractures | 146 (40%) | 135 (39%) | 11 (52%) | 0.2556 |
| FFI* | 1.29 ± 0.22 | 1.26 ± 0.19 | 1.77 ± 0.15 | < 0.0001* |
| angulation in the a.p. radiograph (°) | 6 (2,11) | 6 (1,11) | 12 (8,14) | 0.0003 |
| angulation in the lateral radiograph (°) | 17 (14,24) | 17 (14,24) | 20 (14,28) | 0.309 |
| angle of fracture line in the a.p. radiograph (°) | 8 (0,15) | 7 (0,14) | 17 (0,34) | 0.0112 |
| angle of fracture line in the lateral radiograph (°) | 14 (0,25) | 13 (0,25) | 33 (21,43) | < 0.0001 |
| Number of patients with translation in the a.p. radiograph | 132 (36.1%) | 125 (36.2%) | 7 (33.3%) | > 0.9999 |
| Number of patients with lateral translation in the lateral radiograph | 107 (29.2%) | 104 (30.1%) | 3 (14.3%) | 0.1434 |
| Number of patients with shortened fractures | 74 (20.2%) | 70 (20.3%) | 4 (19%) | > 0.9999 |
| Number of patients with oblique fractures (> 30°) in the a.p. radiograph | 24 (6.6%) | 17 (4.9%) | 7 (33.3%) | 0.0001 |

**Table S1.**

Demographic data and fracture characteristics of our cohort of 366 patients with diametaphyseal radius fractures. Metric data are presented as median with interquartile ranges in brackets as data is not normally distributed; *, values are presented as mean with standard deviation as data is normally distributed; **, p-values are calculated using the U-test for metric data not normally distributed and using the t-test for metric data distributed normally; for categorical variables the Chi-squared test was used; DMRFs(-), diametaphyseal radius fractures defined according to Lieber et al.; DMRFs(+), diametaphyseal radius fractures considered diaphyseal by Lieber et al. but still considered DMRFs by our definition (Lieber et al., 2010); °, angle in degree;


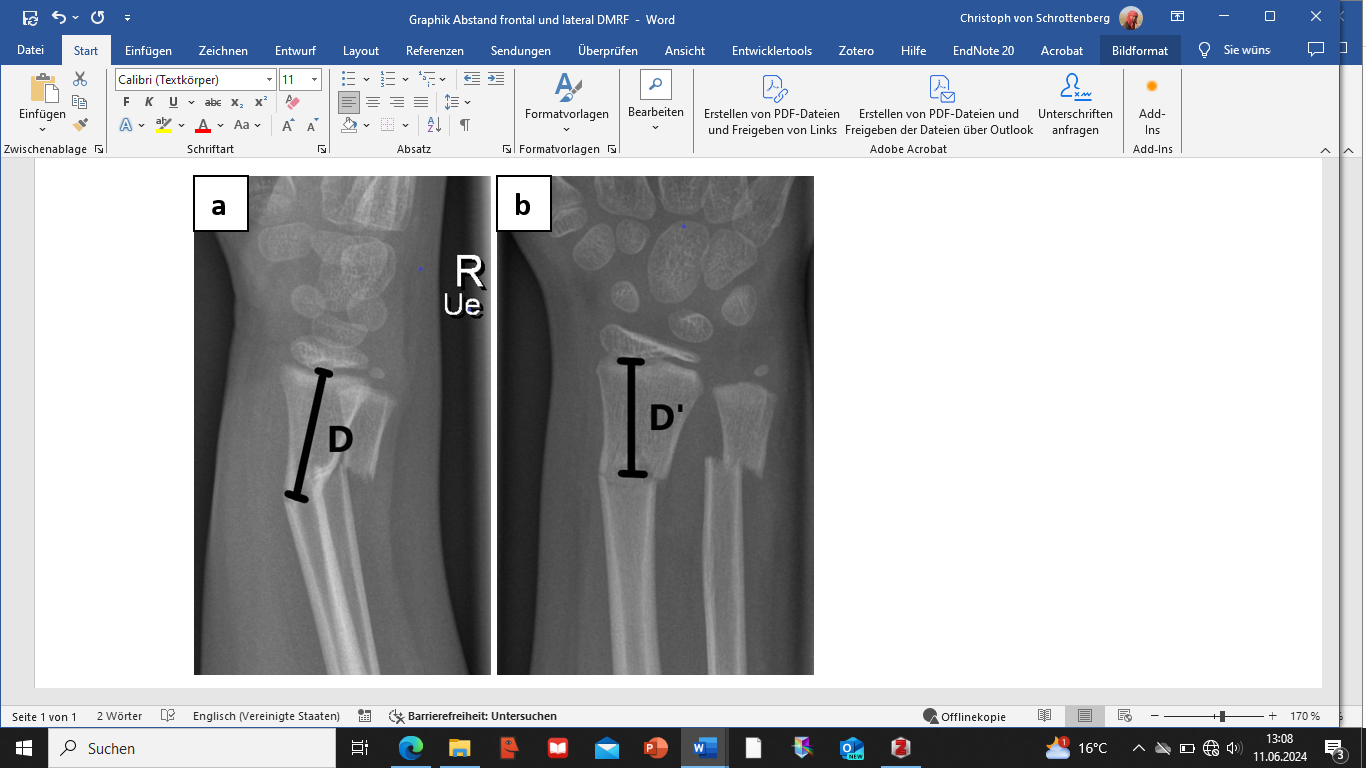


**Figure S2.**

**a.** The actual fracture’s distance to the radius growth plate assessed in the lateral radiograph (D); **b.** The fracture’s assumed distance to the radius growth plate assessed in the a.p. radiograph (D’); D and D’ are not the same (**D ≠ D’**), as the angulation of the distal fragment leads to a mistakenly shorter measurement of the fracture’s distance to the radius growth plate in the a.p. radiograph;
